# Lecture upon Sulphur

**Published:** 1887-12

**Authors:** J. T. Kent


### LECTURE UPON SULPHUR.

PROFESSOR J. T. KENT, A. M., M. D.

(Continued from page 406.)

We often find grand indications for the use of Sulph. in the delay of the first menses. Glands slow of development; unhealthy skin; pimples upon the face; languid; tired; seventeen or eighteen years of age; menses not yet appeared, or scant in flow. Puls. and Sep. are also of great value in this condition. In the leuco-phlegmatic temperament, with damp, cold feet, damp and pallid skin, Calc. follows well after Sulph.

Dysmenorrhœa. A strong indication for its use in this trouble would be, the flow has appeared attended by great pain, which is *agg.* by bathing during the week of menstruation.

Related to this condition, we have another remedy of great value in Calc. phos. A woman having reached the age of twenty-five or thirty years, and having suffered from dysmenorrhœa since the first menstrual flow, caused by lack of development—Stenosis.

Mothers should teach their daughters the fatal consequences following undue exposures and over-exertion at these periods, and should be taught that with improper treatment those evils follow them a lifetime, to the utter ruin of their physical well being.

Sulph. promotes the expulsion of moles, the false or blighted conceptions to which a broken-down physical condition gives rise.

An imperfect development of a fœtus, even though it occur but once, is an indication for the administration of this remedy.

We have spoken of Apis and its efficiency in threatened abortion. Followed by Sulph. it cures the predisposition to abort at future pregnancies.

Puls., Hell., Aletris., Cauloph., are used to prevent this tendency, but none are so frequently indicated as Sulph.

Give a new-born babe a dose of Sulph., it will prevent many troubles. It is well you should look into and study these things that you may provide for and prevent many conditions that may arise.

Should father or mother be syphilitic, give a few doses of Merc. Should either be sycotic, give a dose of Thuj.

In vaccination, one dose of Sulph. before or after the completion of the vesicle. Of course, this is when you do not see indications for another remedy.

Many expert homœopaths give one dose of Sulph. during or after completion of pustulation.

Many give one dose of Sulph. two weeks or a month before a dose of Thuj., because of the known sycotic action of Thuj. Vaccininum is also used.

In the general secondary conditions attending puerperal fever with sepsis, you will find Sulph. a leading remedy.

Many undertake to give Veratr., Arn., Acon., because of the impending inflammation, and because these remedies correspond to the first stage of fever. Dunham says, "Why not go to the root of the fever at once, and give Sulph.," as in ninety-nine cases out of a hundred Sulph. will have cured the fever within twenty-four hours, and the pulse will be again normal. If you treat it in this way, you will have few losses and find it as simple as measles.

Given violent chills, rigors, rapid pulse, fever, even delirium, and in twenty-four hours you can relieve the whole thing—not with the 2x or 3x, or with Quinine. Experience is a good teacher, and *I know.*

In threatened suppuration of the mammary gland, when the lump first appears, one dose of Sulph. may abort the whole trouble. Remedies commonly resorted to to assist suppuration are Merc., Hep., Sil.

A lump in the throat, hard, sticking pains in the ears, *agg.* at night, *agg.* by heat of the bed or application of heat, clammy sweat, inclination to chill and collapse, Merc.

In addition, foul breath, loaded tongue, disordered stomach; still Merc.

Should this condition change, or in the beginning should the pain be relieved by heat, and suppuration be inevitable, Hep. will hasten suppuration. Hep. comes between Merc. and Sil., and both follow Hep.

Sil. materially hastens suppuration, easing the pain and mollifying the death of tissue. Felons forming, suppuration inevitable—Sil. will open in a night, and the pains will be eased in a few hours. Facts that now look so impossible, you will see many times in a well-indicated remedy.

We have many valuable points under breathing, cough, and lungs. Dry, hacking, teasing coughs; great emaciation of the chest; scant expectoration (Sulph. also has copious expectoration, a muco-purulent sputa); chronic hoarseness (a hoarseness from collection of mucus in the larynx), and competes with Caust., Phos., and Carb. veg. Caust. has aphonia, a paralytic condition of the vocal chords. A case in clinic, not spoken aloud for years, cured by Dr. B. Pind, with Caust.[3m].

Difficult breathing; suffocation; wants doors and windows open; wants cool air; cannot endure the close, warm room. You will find almost any kind of expectoration, any kind of cough, with this emaciation of the chest. Chronic conditions which are the result of pneumonia, where the patient had before been troubled with a chest complaint, and in which we find slow resolution, lingering cough with expectoration, emaciation and night-sweats (not tuberculosis), yet threatened with this cachectic state, ultimately ending in phthisis. Sulph., Phos., Lyc., best correspond to these conditions. If you find the mental symptoms of Sulph., with burning of soles and palms, all gone feeling at eleven A. M., wants doors and windows open, great emaciation about chest, it will be speedily relieved by that remedy.

You will be astonished at its working in the last stage of hepatization in pneumonia; prostration great; not doing well; sweats a great deal; likes to be kept cool; if allowed may go down, or may improve slowly. This remedy induces resorption.

Now, there is a condition—would I could picture it to you—when good prescribers frequently lose their patients by prescribing Ars. It is a state of collapse; the patient is low, *very* low; excessive exhaustion; hardly able to raise the hand to the head; there has been great thirst; great restlessness; but now he is too prostrate to move. One single dose of Ars. relieves; but should you not follow it the next morning with Sulph., your patient *will*

*die.* Ars. brings about the tendency to reaction, Sulph. the tendency to resorption. The proper dose at the proper time cures the case. Repeat Ars., and it will overwork, and you might better have given nothing. In all grave cases the second prescription must antidote the first. Ars. and Phos. have a similar relation, and have been so used. Your reputation as prescriber is made on such cases as these. Any old woman may cure a cold.

Exudation after pneumonia, pain in chest. Compare with Puls. and Bry.

Surging of blood from left side of chest to head; feels like flashes of heat surging to the head; rising sensation of heat to the head; head feels hot and flushed; faint feeling from chest up into neck, face, and head. In checking an inflammatory process, Sulph. induces resorption.

In caries of the vertebræ, Calc. and Phos. compete.

Rheumatic, drawing, tearing, lancinating pains in the joints and the limbs, with cramps of the muscles; cold hands and feet, or they may be very hot, alternating; there is a red-string symptom, "soles cold and sweating," somewhat like Merc.; pains *agg.* by heat of bed or from sweating; lower extremities numb, prick and go to sleep; nervous irritation, even in children; child jumps up and cries out.

While chorea and chronic spasms are not as common to it as to some other remedies, nevertheless it goes to the cause and relieves the condition. Agar. and Mygale relieve temporarily, and sometimes cure permanently. Sulph. makes a beginning and sometimes cures wholly. After a suppression the remedy would be Sulph.

Fainting fits alternating with hot flashes; sinking feeling, most likely to come on by the evening or at night.

You will rarely cure a case of epilepsy with Sulph. You may postpone the fits for a few months—Sulph. indicates the cause. You will hardly cure epilepsy every time, you can modify it, and you won't cure the same disease in different individuals with same remedy.

Sulph. has a great amount of trembling and weakness. Sleep symptoms are important: always unrefreshing; wakes in the morning quite tired out; sleeps too soundly; goes to sleep early in the evening, sleeps until midnight; lies awake until daylight; sleeps and can hardly be wakened.

Here we have a resemblance to Nux v. Nux v. patient will go to sleep early in the evening and sleep until three A. M. The sleep of these two remedies is well worth the knowing, especially in their relations to hemorrhoids.

When the patient is sensitive to the actions of a remedy, Nux v. is well indicated.

Where the apparently indicated remedy refuses to act, a dose of Sulph. is needed.

In a last stage of fever not septic.

In a first stage of sepsis.

In convalescence from fevers, where there is general sluggishness, slow reaction, great emaciation.

Its skin symptoms are very important.

In the typical itch there is a slight itching, which demands relief from scratching; relief does not come; burning and numbness set in; little, hard lumps appear under the skin; finally cool, and the itching stops. Skin symptoms and rheumatic pains *agg.* by heat; drawing pains *agg.* by heat of bed; skin symptoms *agg.* by wet.

Sulph. will usually clear up the condition that produces boils.

Sulph. is the chronic of Acon.

Sulph. in a cold climate where ease does not follow the administration of Acon.

Sulph. and Aloes complementary and antidotes to each other.

Sulph., Nux v., and Aloes are complementary to each other and antidotes to each other.

